# The Dilemma of Pneumatosis Intestinalis with Pneumoperitoneum: Nonoperative or Surgical Management—Analysis of a Case

**DOI:** 10.1155/2013/564385

**Published:** 2013-04-04

**Authors:** A. Rossetto, M. Brizzolari, E. Scarpa, G. Terrosu, V. Bresadola

**Affiliations:** Department of General Surgery, University Hospital of Udine, 33100 Udine, Italy

## Abstract

Pneumatosis intestinalis (PI) is an uncommon condition and can be associated with a wide spectrum of diseases, ranging from life-threatening to innocuous conditions. We report the case of a 46-year-old women coming to our attention for an acute abdominal pain, nausea, vomiting, constipation, and increased inflammatory marks, with a CT showing pneumoperitoneum and pneumatosis intestinalis. The previous diagnosis was advanced neoplasia of unknown origin. Despite the surgical intervention, which excluded an ischemic colitis, the patient died in the early postoperative period. The postmortem diagnosis was carcinoma of thymus gland, and the presence of pneumatosis was put down to metastasis nodes in the pulmonary parenchima. This case demonstrates the wide spectrum of presentation of pneumatosis intestinalis, the importance of a careful radiologic evaluation beside the clinical history, since the identification of correct pathogenesis and treatment can be very difficult.

## 1. Introduction

Pneumatosis intestinalis (PI) is an uncommon condition characterized by the presence of gas within the wall of the gastrointestinal tract. PI is a physical or radiographic finding, associated with a wide spectrum of diseases, ranging from life-threatening to innocuous conditions. For this reason, the PI management can vary from urgent surgical procedure to nonoperative management. In only 15% of the cases, PI is defined idiopathically, since it is not related to a specific disease [1–5]. This paper reviews the case of an oncologic patient with a PI and findings of acute abdomen in order to stress, more generally, the difficulties in the identification of correct pathogenesis and consequent treatment [3, 5, 6].

## 2. Case Report

**Figure 1:**
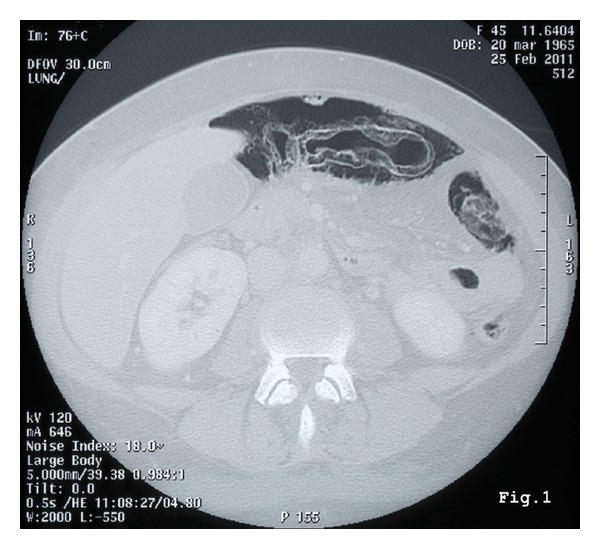
CT of the abdomen, signs of pneumoperitoneum, and pneumatosis.

**Figure 2:**
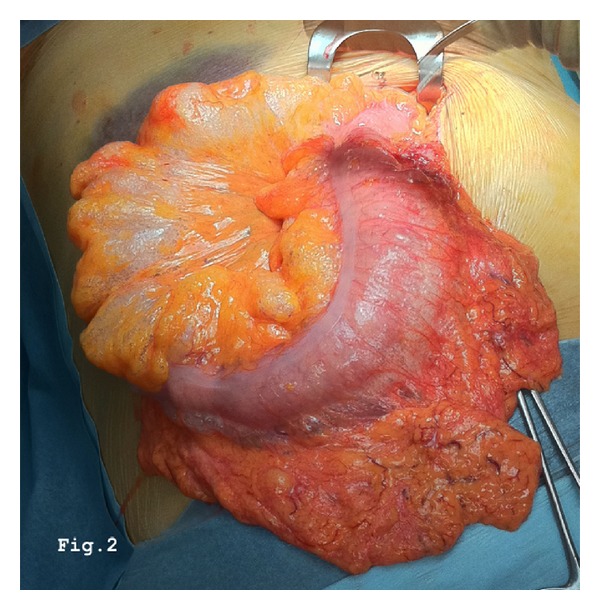
Intraoperative findings: pneumatosis with air bubbles in the mesentery and in the intestinal wall.

**Figure 3:**
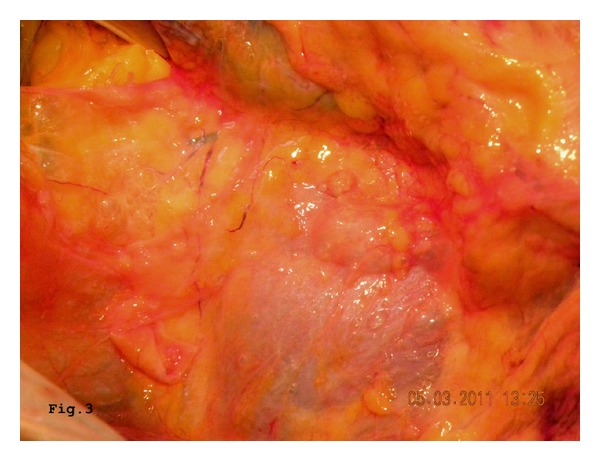
Autopsy: air bubbles in the mediastinum reaching the retroperitoneal space.

We report the case of a 46-year-old women hospitalized at the Department of Oncology with a diagnosis of advanced neoplasia of unknown origin. The diagnostic workup by means of total body CT scans followed from a PET-CT had shown disseminated neoplasia in the lungs, right kidney, and adrenal gland and in the 8th hepatic segment. In addition, a prominent lymphadenopathy, compatible with metastasis, was described in the cervical area, the mediastinal space, and along the aortic caval space, without interruption. A cervical lymph node biopsy revealed a metastasis from a poorly differentiated carcinoma with some aspects of sarcoma probably of kidney origin. Awaiting a systemic chemotherapy, the patient was transferred in our surgical unit because of an acute abdominal pain with signs of peritonitis on physical examination. Nausea, vomiting, and constipation were associated. Inflammatory marks were increased: white blood cells 26380/ul, C-reactive protein 243 mg/dL, and erythrocyte sedimentation rate 50 mm/h. A new abdominal CT scanning showed pneumoperitoneum, intramural air in the colon, and gas collection in the mesentery (Figure 1). On these findings, we made a differential diagnosis between a “simple” PI and an intestinal perforation. On the clinical, laboratory, and radiological findings we opted for an explorative laparotomy. Celiotomy showed pneumatosis along most of the colon, but no perforation was identified. The presence of gas collection with air bubbles in the mesentery and in the retroperitoneum was also confirmed (Figure 2). The exploration of the abdomen confirmed the presence of neoplastic lesions, previously described by PET-CT, associated with peritoneal carcinosis. After colostomy, a few biopsies of the mucosa and the bowel wall were performed in order to exclude an ischemic colitis. In both cases the macroscopic appearance was regular. After 6 days in intensive unit, the patient died due to poor general condition and the onset of a multiorgan failure. The autopsy confirmed the situation of advanced cancer and the presence of multiple gas-filled cysts protruding through the colon wall, mesentery, and retroperitoneum. Unlike the surgical and CT scanning reports, the presence of air bubbles was also detected in the mediastinum, occupied by metastasis nodes in the pulmonary parenchyma (Figure 3), explaining the origin of pneumoperitoneum. After immunohistochemical study of cancer specimens by means of cytokeratin 18 and vimentin, the final diagnosis was carcinoma of thymus gland with sarcomatoid characteristic (T4N3aM1, IV stage in according with WHO 2004).

## 3. Discussion

PI is an uncommon condition which has recently arisen to clinical attention thanks to improved radiographic identification, with an incidence of 0.37% in patients submitted to abdominal CT scans [2]. The actual PI incidence is unknown due to its frequent asymptomatic course. PI can be subdivided into two groups: primary, 15% of cases, a benign idiopathic condition, usually asymptomatic and incidentally found and secondary, 85% of cases, including necrotic bowel disorders, nonnecrotic bowel disorders, infective causes, and pulmonary causes [6–8].

 The conditions underlying secondary PI can be divided into six groups: traumatic and mechanical, inflammatory and autoimmune, infectious, pulmonary, drug induced, and others (as the necrotizing enterocolitis) [1]. Multiple theories have been discussed for the pathogenesis of PI. At present three possibilities have been put forward in order to explain the source of gas in the intestinal wall: mechanical, bacterial, and pulmonary. According to the mechanical theory, the gas intrusion into the bowel wall is caused by a mucosa injury (inflammatory process, defect in the immune barrier of the gut, steroid, or cytotoxic therapy) and often on an increased intraluminal pressure (endoscopic procedure, intestinal obstruction). The bacterial theory argues that the gas produced by gas-fermenting organisms infiltrates the bowel wall through an injury of the mucosa or because a gradient between intraluminal and serum partial pressures, while, for the pulmonary theory, air could come from the thoracic cavity through retroperitoneum [4].

This radiological finding can be caused by a number of conditions, and the extent of the PI does not correlate with the severity of the underlying disease but with its localization and the surgical treatment in many cases is not required, while it can be mandatory if the underlying cause of PI is perforation or bowel ischemia. Therefore the most effort should be made in identifying the underlying cause, searching for pneumoperitoneum or portal venous gas. The decision for surgical intervention should be secondary to a very accurate study of the patient's history beside the clinical presentation, since there are so many conditions which can cause this radiological finding but do not require any surgical treatment [6, 7].

Khalil et al. proposed a very interesting management algorithm for pneumatosis in which the presence of critical CT findings, critical laboratory findings, conspicuous physical conditions, or examination all lead to the surgical exploration [5]. Otherwise, in presence of a conspicuous medical history, reevaluation is suggested after treatment of the underlying disease [8–16].

We completely agree with this suggestion, even if in our case, possibly we might have not performed surgical treatment without any difference in the clinical outcome. The clinical conditions on admission and the clinical laboratory findings were so poor, that the radiological finding of gas simply confirmed a strong preoperative suspicious. Moreover, our preoperative CT detected, along with the presence of gas on the colon wall, the presence of pneumoperitoneum, which in absence of previous trauma or iatrogenic endoscopic trauma makes the surgical approach absolutely mandatory.

The presence of unknown neoplasia, even considering lung metastasis, made it more difficult to hypothesize the underlying etiology of pneumoperitoneum.

In this case autopsy revealed a very strange origin of pneumatosis highlighting the complex wide spectrum of presentation of PI. We believe that our study confirms as mandatory steps in the management of such cases the most meticulous radiological evaluation through CT scanning together with careful evaluation of the medical history and the clinical presentation of the patient.
